# Case Report: Successful treatment of guselkumab in a patient with verrucous psoriasis unresponsive to secukinumab

**DOI:** 10.3389/fimmu.2025.1596544

**Published:** 2025-11-04

**Authors:** Jianfeng Zheng, Xuemei Yi, Yuling Shi, Yangfeng Ding

**Affiliations:** Department of Dermatology, Shanghai Skin Disease Hospital, Tongji University School of Medicine, Institute of Psoriasis, Tongji University School of Medicine, Shanghai, China

**Keywords:** verrucous psoriasis, plaque psoriasis, secukinumab, guselkumab, case report

## Abstract

A 77-year-old Chinese male with a 10-year history of plaque psoriasis had recurrent episodes of symmetric hypertrophic verrucous plaques in the lower legs. The patient was monitored for the evolution of the disease over two years before he came to our attention. Because the lesions were resistant to topical glucocorticoids and vitamin D3 at another dermatological center, the patient was treated with secukinumab for one year. However, the verrucous lesions further worsened, and the patient visited the outpatient department of our hospital in March 2023. Treatment with guselkumab was started. It immediately attenuated the plaques of the trunk and limbs. Surprisingly, the verrucous plaques of both legs showed complete resolution in 6 months.

## Introduction

Verrucous psoriasis (VP) is a scarce variant characterized by a bizarre, papilloma-like appearance with wart-like thick scales (1). Although the etiology remains unclear, it develops from pre-existing psoriasis and may be associated with varicose veins in the lower extremities or dysfunction of metabolic conditions such as obesity and diabetes mellitus (2). There is a higher prevalence in men (3). VP is often resistant to therapy. Treatment options are limited and based on published case reports which include topical and systemic agents (4). Here, we report a case of VP in the lower legs superimposed on pre-existing psoriatic plaques, who was treated with guselkumab.

## Case report

**Figure 1:**
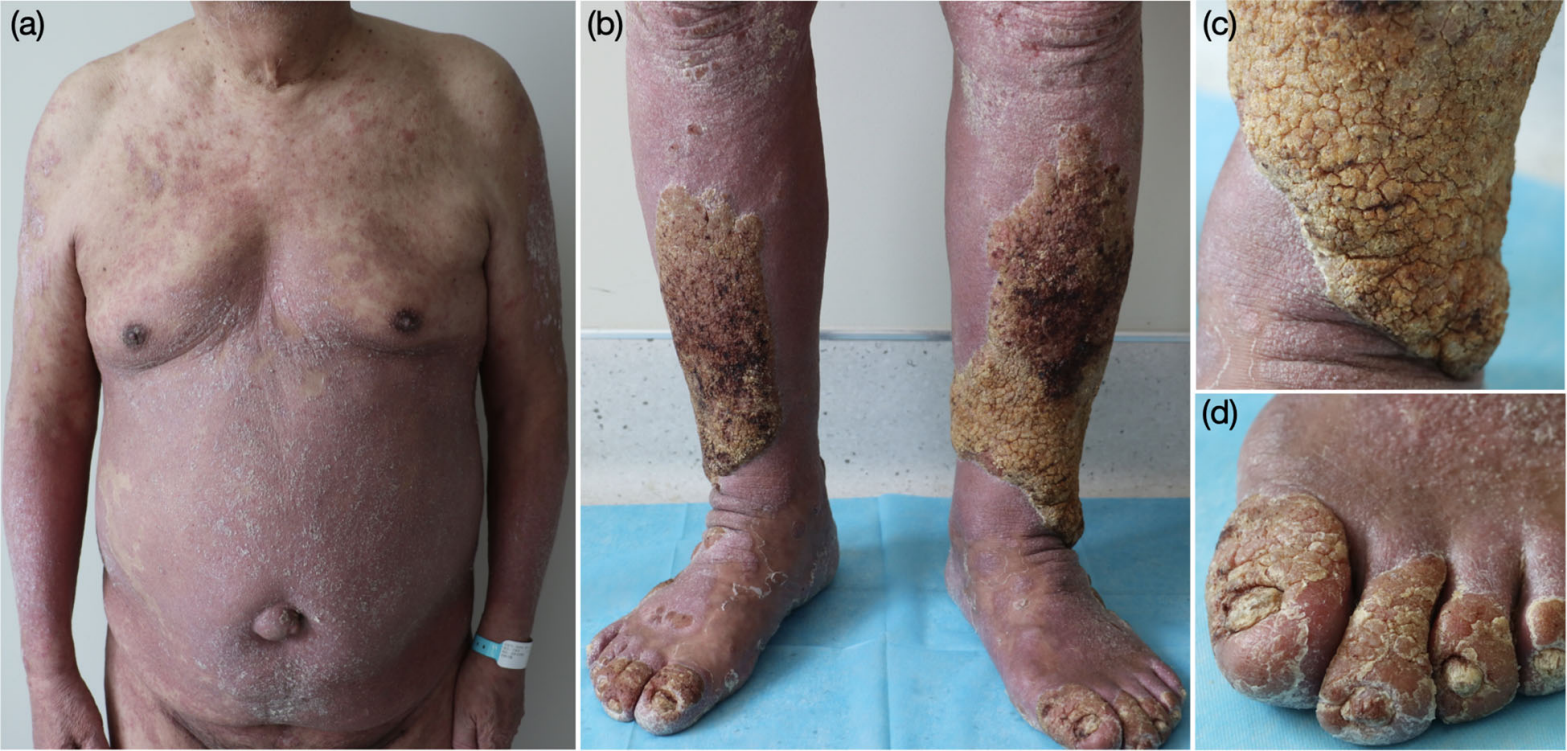
Clinical features of the patient at the first visit. **(a)** erythematous plaques of the trunk, **(b–d)** aggregation of multiple extensive verrucous, yellowish–gray plaques with a scaly surface on erythematous plaques on both lower legs.

A 77-year-old Chinese male with a 10-year history of plaque psoriasis had recurrent episodes of symmetric hypertrophic verrucous plaques of both lower legs superimposed on pre-existing psoriatic plaques. The patient was monitored for the evolution of the disease over two years before he came to our attention. In 2022, his lower legs had become swollen and covered with thick scales. The lesions gradually became cobblestone-like and continued to worsen despite the administration of secukinumab for 1 year at another dermatological center. Therefore, the patient visited the inpatient department of our hospital in March 2023. This patient showed symmetric hypertrophic verrucous plaques of both lower legs accompanied by erythematous plaques affecting 76% of the whole body (**Figures 1a–d**). The patient’s psoriasis severity index (PASI) score was 33.5. He had a 10-year history of hypertension, a 10-year history of prostate hyperplasia and mild obesity (body mass index, 26.4).

**Figure 2:**
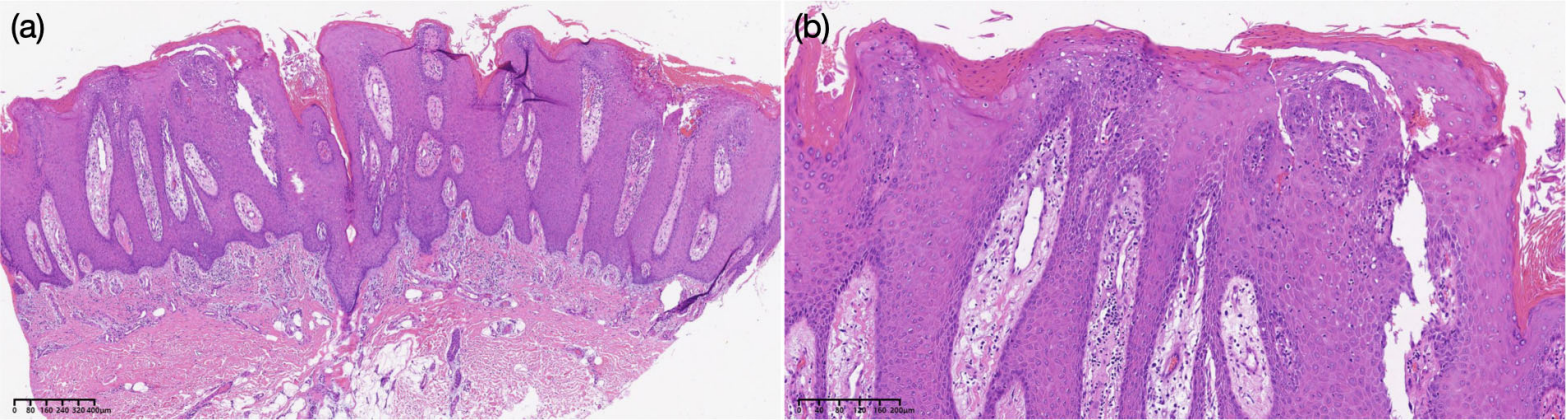
Histopathological findings of the nodule of the left leg [hematoxylin–eosin, original magnifications: **(a)** *20, **(b)** *200].

Before starting treatment, a comprehensive set of laboratory and instrumental tests, including chest X-ray, electrocardiogram (ECG), complete blood count, complete liver profile, creatinine, antinuclear antibody, acquired immunodeficiency syndrome screening, rapid plasma regain test, hepatitis B virus serologic markers, hepatitis C virus antibody, and tuberculosis assay, yielded negative results, except for C-reactive protein (31.10 mg/L) erythrocyte sedimentation (23 mm/h), and total IgE (108 IU/L). Moreover, staphylococcus aureus and proteus mirabilis were detected on verrucous lesions. A biopsy from a verrucous lesion of the left leg revealed hyperkeratosis, neutrophil infiltration in the corner layer, wart-like hyperplasia of the epidermis, microabscess formed by the accumulation of neutrophils in the spinous layer (**Figures 2a, b**), PAS staining (–), anti-acid staining (–).

Based on the clinical and pathologic features, we started treating him with oral anti-psoriatic Chinese medicine, clindamycin, topical vitamin D3, and antibiotics, none of which, however, resulted in a favorable response on verrucous lesions. Two weeks after admission, the patient was administrated a dose of 100 mg guselkumab. Strikingly, soon after guselkumab was introduced, the plaque psoriasis on his trunk and limbs became flattened, and VP on both lower legs regressed. Unfortunately, the patient chose to interrupt the follow-up treatment.

**Figure 3:**
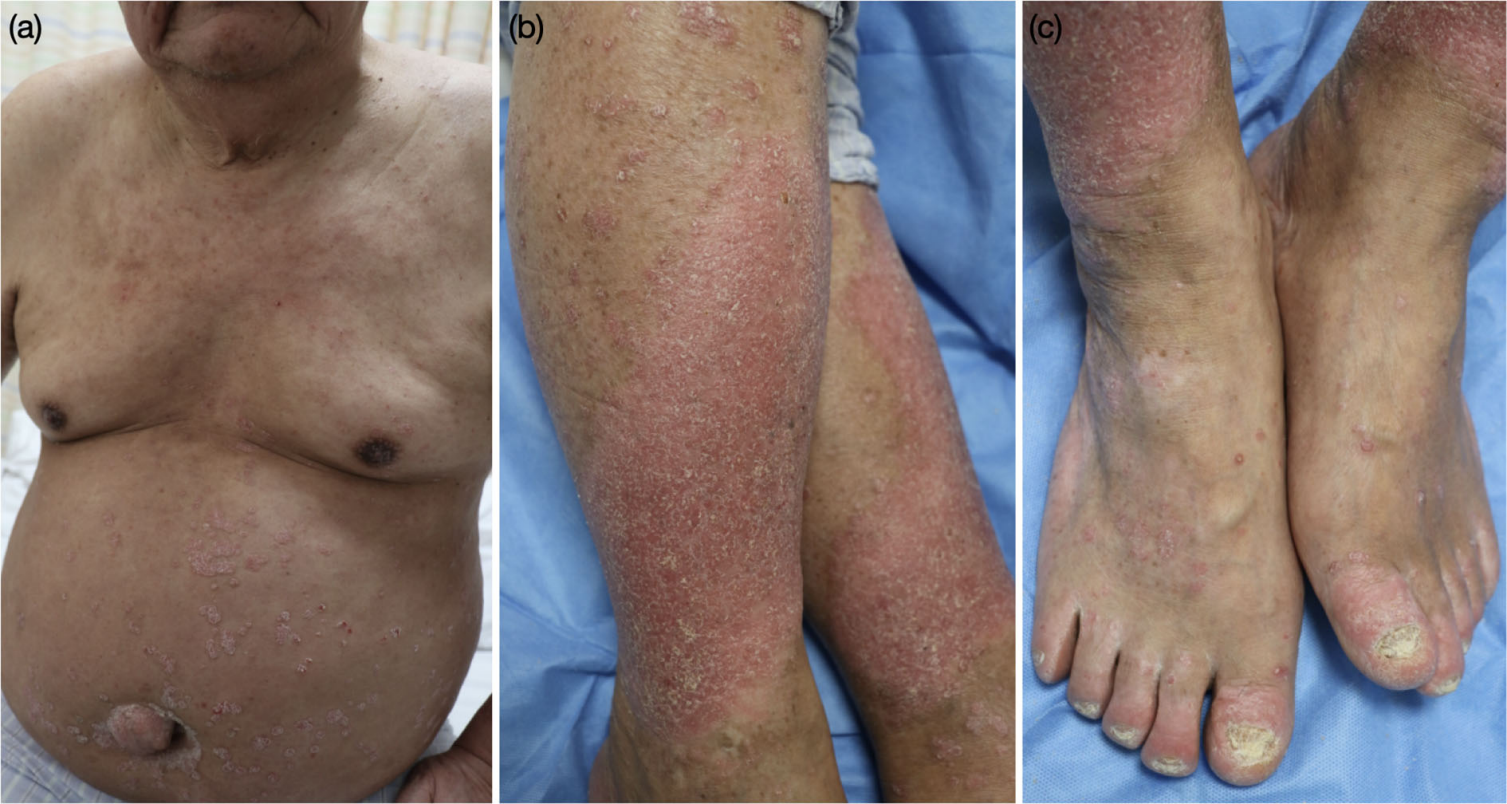
Clinical features of the patient at the second visit. **(a–c)** erythematous papules and plaques of the trunk and limbs.

6 months after his first treatment, we conducted our telephone follow-up of the patient, and the verrucous plaques of both legs showed complete resolution. In January 2024, due to the recurrence of plaque psoriasis, the patient visited the inpatient department of our hospital again. Physical examination revealed the presence of erythematous papules and plaques which involved the trunk and extremities (**Figures 3a–c**). The patient’s PASI score was 13.0, so he was given the second dose of 100 mg guselkumab.

## Discussion

Guselkumab represents an anti-interleukin (IL)-23 monoclonal antibody that reduces inflammation in psoriatic lesions by directly inhibiting IL-23/Th17 signaling (5). It is approved for treating moderate-to-severe plaque psoriasis in cases eligible for systemic therapy or phototherapy (6). As we know, the IL-23/IL-17 axis is considered to play an important role (5). Particularly, IL-23 is a dimer comprising a specific subunit, p19, and a p40 subunit, also found in IL-12. These cytokines may activate two T-cell types, including helper T-cell types 1 and 17, which release psoriatic cytokines such as IL-17, interferon-γ, TNF-α, and IL-22 (7). This may explain why some patients previously administered anti-IL- 17 still showed an excellent response to guselkumab.

Diagnosing VP can be complex due to its similarity in clinical presentation and histopathology to other conditions, such as verruca vulgaris, epidermal nevus, squamous cell carcinoma, hypertrophic lichen planus, discoid lupus erythematosus, eczema, and fungal infection (8). In our case, the patient had shown symmetric hypertrophic verrucous plaques of both lower legs superimposed on pre-existing psoriatic plaques for two years, which had faced a diagnostic challenge. A diagnosis of VP was established based on the clinical and pathologic features after the patient visited the inpatient department of our hospital.

Regarding treatment, more extensive clinical studies of high methodological quality are not available due to the overall rarity of VP. Based on a limited number of case reports, published review articles have suggested multiple therapies including topical ointments, methotrexate, systemic retinoids, cyclosporin A, and biologics. However, their success rates are inconsistent. Therefore, there is a lack of standardized treatment guidelines for VP. Zhixuan Guo et al. (9) reported that a 61-year-old male patient had experienced significant improvement in his skin lesions upon starting secukinumab. However, the thick verrucous plaques on the patient’s limbs and trunk emerged after a year of secukimumab administration. Similarly, our patient was treated with secukinumab for one year. However, the verrucous lesions further worsened. In 2024, Artur Antonio Duarte et al. (10) reported that a 31-year-old female patient with verrucous psoriasis showed an excellent response to Adalimumab, reaching PASI 90 after 12 weeks of treatment. Luckily, our patient showed complete resolution 24 weeks after the first treatment.

So far, the pathology of VP has not been fully understood. Notably, our patient experienced pruritus, elevated IgE levels, and histological evidence of focal interface dermatitis, suggesting an immune-mediated reaction potentially aggravated by the immuno-modulatory effects of secukinumab. Furthermore, a recent report showed that apremilast could be documented as an effective agent in the treatment of VP (11). These findings underscore the indispensable role of inflammatory pathways in the regulation of keratinocyte differentiation.

## Conclusion

To the best of our knowledge, this marks the inaugural utilization of guselkumab in the treatment of VP. Our case underscores the efficacy of guselkumab for VP and advocates its consideration in instances of conventional treatment-resistant VP. While specific studies have indicated the involvement of type 2 inflammation in VP pathogenesis, further clinical and foundational research is imperative to corroborate this conclusion.

## Data Availability

The raw data supporting the conclusions of this article will be made available by the authors, without undue reservation.
